# Associations of alcohol use with expression of stromal markers in benign breast biopsy samples

**DOI:** 10.1038/s41416-026-03481-3

**Published:** 2026-06-11

**Authors:** Anjoli Armstrong, Yujing J. Heng, Brian R. Sardella, Maisey Ratcliffe, Bernard Rosner, Rulla M. Tamimi, Lusine Yaghjyan

**Affiliations:** 1 University of Florida, College of Public Health and Health Professions and College of Medicine, Department of Epidemiology, Gainesville, FL USA; 2 Department of Pathology, Beth Israel Deaconess Medical Center and Harvard Medical School, Boston, MA USA; 3 Channing Division of Network Medicine, Department of Medicine, Brigham and Women’s Hospital and Harvard Medical School, Boston, MA USA; 4 Department of Population Health Sciences, Weill Cornell Medicine, New York, NY USA; 5 Cancer Prevention and Control Research Program, Sandra and Edward Meyer Cancer Center, New York, NY USA

**Keywords:** Breast cancer, Biomarkers

## Abstract

**Background:**

We explored the associations of alcohol consumption with expression of α-Smooth Muscle Actin (α-SMA), Tenascin-C (TNC), Fibroblast Activation Protein (FAP), Matrix Metalloproteinase-14 (MMP14), and Calcyclin (s100a6) stromal markers in benign breast biopsy samples.

**Methods:**

The study included 683 cancer-free women from the Nurses’ Health Study II who had biopsy-confirmed benign breast disease (BBD). Alcohol consumption was assessed with semi-quantitative Food Frequency Questionnaires. The data on breast cancer (BCa) risk factors were obtained from biennial questionnaires. Immunofluorescence for stromal markers was performed on tissue microarrays. For each core, the % positivity was quantified by inForm v2.6.0. Generalised linear regression was used to examine associations between alcohol consumption (recent [at biopsy date] and cumulative average from all available questionnaires before the biopsy date) and log-transformed expression of each marker, adjusting for BCa risk factors and BBD subtype.

**Results:**

In multivariate analysis, we observed a suggestive positive association of cumulative average alcohol with TNC (*β* per 11 g/day = 0.59, 95% CI −0.03,1.22, *p* = 0.06). Alcohol consumption was not associated with α-SMA, FAP, s100a6, and MMP14.

**Conclusion:**

Alcohol consumption may be associated with an increased normal stromal fibroblast activation as measured by TNC expression in histologically normal breast tissue. Future studies are warranted to confirm our findings.

## Introduction

Alcohol consumption is a well-established, modifiable breast cancer risk factor with numerous studies reporting positive associations [1–3]. Women who drink 15–30 grams of alcohol per day (about 1–2 drinks) are at a 10–40% increased risk of breast cancer compared to non-drinkers, and each additional drink per day increases the risk by about 7% [4–7]. In addition, research shows that although alcohol consumption increases breast cancer risk among pre- and postmenopausal women, postmenopausal women may face a slightly greater risk [3]. Importantly, the recent report from the US Surgeon General emphasises that the associations of alcohol with breast cancer are causal [8].

Various mechanisms may explain the influence of alcohol consumption on breast cancer risk. As part of the oxidative metabolism pathway, ethanol is converted to acetaldehyde, which forms adducts with DNA, leading to disruption of DNA synthesis and repair and inducing mutations [9, 10]. Importantly, compared to the liver, breast tissue significantly differs in its ability to detoxify acetaldehyde, resulting in its accumulation and increasing toxicity in this environment [2, 10]. Other mechanisms by which alcohol can influence breast cancer risk include oxidative stress and inflammation, as well as changes in metabolism of oestrogens [9, 10].

In addition to breast cancer risk, in some previous studies, alcohol consumption was also positively associated with mammographic breast density, one of the strongest risk factors for breast cancer [11–13]. Mammographic breast density is reflective of the proportion of stromal, epithelial, and adipose tissues in the breast. The stroma contributes greatly (approximately 30%) to the variation in breast density vs. only 4–7% explained by epithelium [14, 15]. Importantly, emerging evidence has highlighted the active role of stroma in tumorigenesis via various mechanisms. Normal (resident) fibroblasts are the most abundant stromal cells in the tumour microenvironment (TME), primarily responsible for extracellular matrix synthesis and remodelling and creating and maintaining connective tissue [16]. In addition to their involvement in important regulatory pathways, normal breast fibroblasts can give rise to cancer-associated fibroblasts (CAFs) [17, 18]. Whether alcohol can influence the activity of normal fibroblasts is unknown. To fill these gaps, we examined the associations of alcohol consumption with α-Smooth Muscle Actin (α-SMA), Tenascin-C (TNC), Fibroblast Activation Protein (FAP), Matrix Metalloproteinase-14 (or Metallo-peptidase, MMP14), and s100a6 (Calcyclin), all of which are considered to be markers of fibroblast activation. While there have been no studies of stromal tissue markers in normal tissue of cancer-free women, these stromal markers have been studied in breast tumours and adjacent normal tissue and have been previously linked to more unfavourable tumour characteristics and patient outcomes [19–26].

## Methods

### Study population

This study included cancer-free women with incident biopsy-confirmed benign breast disease (BBD) identified within the Nurses’ Health Study II (NHSII) cohort. The NHSII is an ongoing prospective cohort study established in 1989, which enrolled 116,430 female registered nurses aged 25–42 years from across the United States [27]. Information on breast cancer risk factors was collected at the baseline and updated biennially. At baseline and on subsequent questionnaires, participants were asked whether they had ever received a diagnosis of BBD and, if so, whether it had been confirmed by fine-needle aspiration or biopsy. Our study included 683 cancer-free women with biopsy-confirmed BBD with available data on stromal markers, alcohol consumption, and important covariates. The study protocol was approved by the institutional review boards of the Brigham and Women’s Hospital and Harvard T.H. Chan School of Public Health, and those of participating registries as required. Consent was obtained or implied by return of questionnaires.

### Benign breast biopsy confirmation and tissue microarray construction

Women who reported a biopsy-confirmed BBD diagnosis were contacted to obtain permission to retrieve their pathology specimens for review [27]. Hematoxylin and eosin (H&E)-stained breast tissue slides were centrally reviewed by blinded pathologists and classified into one of three histologic subtypes: nonproliferative, proliferative disease without atypia, and proliferative disease with atypia. A study pathologist re-evaluated H&E sections to annotate normal terminal duct-lobular units (TDLUs), which were then used for tissue microarray (TMA) construction at the Dana-Farber/Harvard Cancer Center Tissue Microarray core facility. Up to three 0.6 mm cores of normal TDLU were included in the TMA blocks [28–30].

### Immunofluorescence for stromal markers and image analysis

A 5 µm paraffin section was cut from each TMA block and stained for stromal markers using an automated multiplex immunofluorescence (IF) technique at the University of Florida Pathology Core Lab using the Leica Bond Autostainer according to a previously established protocol (αSMA: Abcam, Cambridge, MA, Cat# ab5694, RRID: AB_2223021, 1:400 dilution; FAP: Abcam, Cambridge, MA, Cat# ab207178, RRID: AB_2864720, 1:50 dilution; MMP14, Abcam, Cambridge, MA, Cat# ab51074, RRID: AB_881234, 1:150 dilution; Tenascin-C, Abcam, Cambridge, MA, Cat# ab108930; RRID: AB_10865908, 1:200 dilution; s100a6, Abcam, Cambridge, MA, Cat# ab181975, RRID: AB_3697229, 1:300 dilution). To distinguish stromal from epithelial compartments, epithelial regions were identified using the cytokeratin marker AE-1/AE-3, enabling accurate segmentation and quantification of stromal staining (panCK AE1/AE3, Novus Bio, Centennial, CO, Cat# NBP2-29429, RRID: AB_3068002, 1:500 dilution).

Using optimised imaging parameters (DAPI 5.12 ms, Opal 480 9.39 ms, Opal 520 52.82 ms, Opal 570 36.79 ms, Opal 620 36.81 ms, Opal 690 30.33 ms, and auto fluorescence 94.89 ms), TMA sections were digitised at ×20 magnification using the Phenolmager (Akoya Biosciences, Marlborough, MA). IF quantification was performed using in InForm v2.6.0 (Akyoa Biosciences). The experienced operator selected seven representative cores in which at least one of the five stromal markers was highly expressed to train tissue segmentation into stromal and epithelial regions based on pan-cytokeratin (Pan-CK) and αSMA expression as well as determine minimum threshold for each marker. Thresholds for marker positivity were set as: PanCK >0.1, FAP >0.25, TNC > 0.25, MMP14 >0.3, αSMA >0.17, and s100a6 >1.

Cell-level data for all detected cores were exported as a .CSV file, and subsequent data processing was performed in R. PanCK-positive cells were excluded from the analysis to eliminate epithelial and myoepithelial populations. SMA-positive cells in epithelial regions were also excluded as these were most likely endothelial cells. The total number of the remaining cells (in the stromal regions) was used as the denominator for downstream quantification.

For each TMA core, marker expression within the stromal compartment was quantified on a continuous scale as the percent of stromal cells staining positive for a given marker, regardless of staining intensity.

### Assessment of alcohol consumption

In NHSII, alcohol consumption was assessed using semi-quantitative Food Frequency Questionnaires (FFQ) in 1989, 1991, and then every 4 years thereafter. Study participants reported their alcohol consumption over the previous year, specifying intake of wine, beer and liquor. Standard drink definitions include: 1 can or bottle of beer, a 4-oz glass of wine, or a single drink/shot of liquor. Participants indicated their drinking frequency using eight categories: 1–3 per month, 1 per week, 2–4 per week, 5–6 per week, 1 per day, 2–3 per day, 4–6 per day, or ≥6 per day. Total alcohol consumption in grams was calculated as the sum of daily drinks multiplied by average ethanol content per drink (12.8 g for regular beer, 11.3 g for light beer, 11.0 g for wine, and 14.0 g for liquor). Recent alcohol consumption was determined based on the questionnaire cycle closest to the date of biopsy and was modelled both as a continuous (per 11 g/day, equivalent to 1 drink) and categorical variable (0 g/day [reference group], <11 g/day, 11–22 g/day, and ≥22 g/day as well as 0 g/day [reference group], <11 g/day, and ≥11 g/day). Cumulative average alcohol consumption was computed by averaging all of a participant’s reported intakes from baseline through biopsy date. It was also modelled as a continuous as well as categorical variable with the above-mentioned approaches. In our study sample, based on the biopsy year, for the majority of participants (75.6%) the cumulative average was calculated based on 4 or more FFQs; for 7.0% it was based on 3 FFQs, and for 17.4% it was based on 2 FFQs.

### Covariate information

Data on breast cancer risk factors were obtained from the biennial questionnaires at the time of the biopsy. Women were considered to be postmenopausal if they reported having no menstrual periods within the previous 12 months (with natural menopause), or having both ovaries removed, or having a hysterectomy, and being 54 years or older for ever smokers or 56 years or older for never smokers [31, 32].

### Statistical analysis

Associations between alcohol consumption and log-transformed expression of five stromal markers (αSMA, TNC, FAP, s100a6, and MMP14) were evaluated using multivariate linear regression. The risk estimates were adjusted for a priori selected breast cancer risk factors: age (continuous, years), age at menarche ( < 12, 12, 13, >13, unknown), BMI (continuous, kg/m^2^), combined parity/age at first birth (parous with first birth before age 25, parous with first birth after age 25, nulliparous, unknown), family history of breast cancer (yes/no), BBD subtype (nonproliferative, proliferative disease without atypia, and proliferative with atypia), and menopausal status/postmenopausal hormone use (premenopausal, postmenopausal/no hormone use, postmenopausal/past hormone use, postmenopausal/current hormone use, and postmenopausal/unknown hormone use status). We additionally examined age and BMI-adjusted models as well as full models without adjustment for BBD subtype. Also, since tenascin had a large number of observations with 0% staining positivity, we additionally used probit transformation to confirm our findings. Median intake values within each alcohol intake category were used for the test of trend. Finally, even though we could not assess the differences in the associations between pre- and postmenopausal women due to the small number of postmenopausal women in our sample, in exploratory analysis we examined associations separately in premenopausal women. All analyses were conducted using SAS software (version 9.4, SAS Institute, Cary, NC, USA). Significance was evaluated at the 0.05 level using two-sided tests.

## Results

**Fig. 1:**
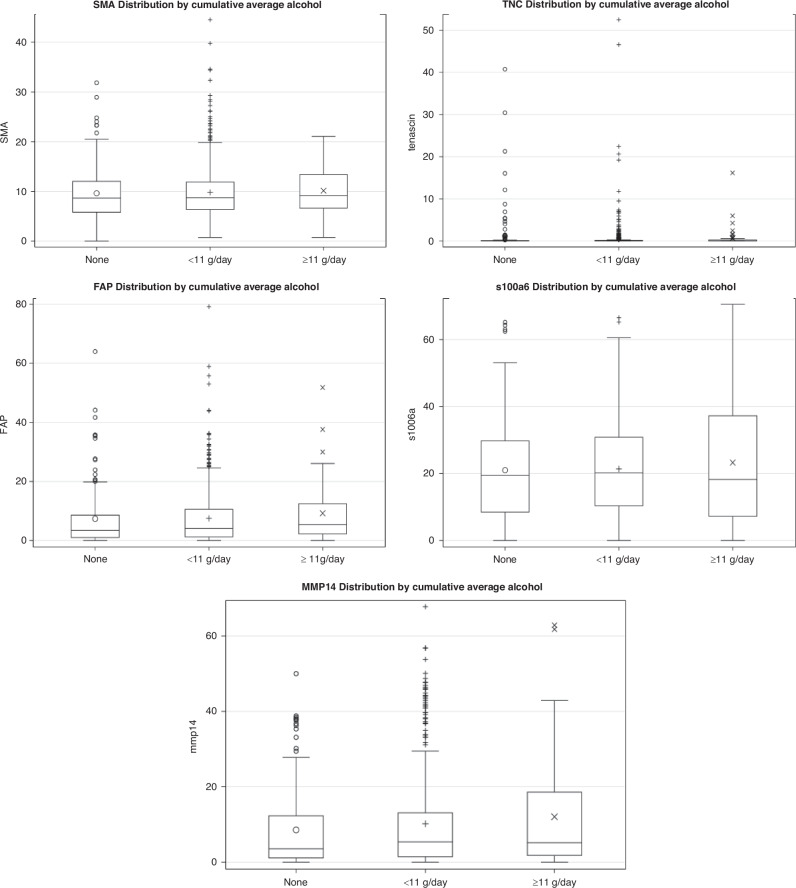
Distribution of stromal markers by cumulative average drinking in the study sample. Each figure represents respective medians, quartiles, and outliers for each stromal marker by the level of drinking (None, <11g/day, ≥11g/day).

Among 683 cancer-free women with incident BBD, the average age at biopsy was 44 years (range of 27–63). The majority of women had proliferative disease without atypia (65.3%), followed by non-proliferative disease (26.2%) and proliferative disease with atypia (8.5%). The majority of women were premenopausal at biopsy (81.3%). Table 1 summarises age-adjusted characteristics of women in the study sample by the level of cumulative average alcohol consumption. In our sample, 150 (22%) women were non-drinkers, 477 (70%) women consumed less than one drink per day ( < 11 g/day), and 56 (8%) consumed at least one drink per day ( > 11 g/day). Distribution of the stromal markers’ expression in our study sample and their correlations with each other are presented in Supplementary Tables 1 and 2, respectively. Distribution of markers’ expression by the level of cumulative average alcohol consumption is presented in Fig. 1. Since tenascin had large number of observations with 0% staining positivity, we additionally present distribution of categorical expression (0, <1, 1–< 10, and ≥10%) (Supplementary Fig. 1) as well as distribution of log-transformed tenascin (Supplementary Fig. 2) by the level of cumulative average alcohol.

**Table 1:** Age-adjusted characteristics of women with biopsy-confirmed incident benign breast disease in the Nurses’ Health Study II, by cumulative alcohol use.

Characteristic	Cumulative average alcohol consumption		
	None *n* = 150	<11 g/day *n* = 478	≥11 g/day *n* = 56
Mean (SD)			
Age at BBD biopsy (years)^a^	44.95 (6.20)	44.21 (6.02)	44.68 (5.84)
Age at menarche (years)	12.44 (1.38)	12.55 (1.36)	12.72 (1.34)
Body Mass Index (kg/m^2^)	26.30 (6.00)	25.50 (5.46)	24.00 (5.29)
αSMA (%)	9.54 (5.32)	9.77 (5.62)	9.68 (4.72)
TNC (%)	1.14 (4.63)	0.66 (3.72)	0.68 (2.26)
FAP (%)	7.05 (9.54)	7.52 (9.49)	9.24 (9.62)
s100a6 (%)	21.27 (15.00)	21.27 (14.18)	24.41 (18.75)
MMP14 (%)	8.64 (10.51)	10.20 (12.33)	14.36 (17.04)
Percentages			
Parity/age at first birth			
Nulliparous	15	13	15
Parous, age <25 years	34	29	25
Parous, age ≥25 years	51	58	59
Family history of BBD	8	10	8
Benign breast disease			
Non-proliferative	24	27	21
Proliferative without atypia	67	65	65
Proliferative with atypia	9	8	14
Smoking status			
Never smoked	77	64	34
Past smoker	17	27	40
Current smoker	7	9	26
Menopausal status/postmenopausal hormone use^b^			
Premenopausal	79	83	81
Postmenopausal/never used	4	4	7
Postmenopausal/past use	2	2	1
Postmenopausal/current use	8	8	2

*αSMA* alpha-smooth muscle actin, *BBD* benign breast disease, *CI* confidence interval, *FAP* fibroblast activation protein, *MHT* menopausal hormone therapy, *MMP14* matrix metalloproteinase, *NA* not applicable, *s100a6* calcyclin, *SD* standard deviation, *TNC* tenascin-C.

^a^Value is not age-adjusted^.^.

^b^The table does not include participants with unknown menopausal status.

Results from the full multivariate and reduced age- and BMI-adjusted models are presented in Table 2 and Supplementary Table 3, respectively. In the multivariate analysis, continuous recent and cumulative alcohol consumption were not associated with the expression of αSMA, FAP, s100a6, and MMP14 (for recent: *β* per 11 g or one drink = 0.03, 95% CI −0.06, 0.13; *β* = 0.16, 95% CI −0.18, 0.49; *β* = −0.05, 95% CI −0.25, 0.14; and *β* = 0.14, 95% CI −0.22, 0.50, respectively; for cumulative: *β* per 11 g or one drink = 0.01, 95% CI −0.11, 0.12; *β* = 0.09, 95% CI −0.29, 0.48; *β* = −0.06, 95% CI −0.29, 0.16; and *β* = 0.06, 95% CI −0.35, 0.48, respectively). Continuous cumulative average alcohol consumption was associated with a borderline significant increase in TNC expression (*β* per 11 g or one drink = 0.59, 95% CI −0.03, 1.22, *p* = 0.06), which was more apparent in the reduced models (*β* = 0.69, 95% CI 0.07, 1.32; Supplementary Table 3). When modelled as categorical (drinks per day), neither recent nor cumulative average alcohol consumption was associated with any of the markers (*p* for trend for all >0.05). However, though within a small strata of heavy drinking in our study sample, heavy cumulative average alcohol consumption ( ≥ 22 g/day [≥2 drinks/day]), was associated with higher expression of TNC (*β* = 2.50, 95% CI 0.20, 4.80) (Table 2), with a similar significant association observed in the reduced model (*β* = 2.65, 95% CI 0.35, 4.95; Supplementary Table 3). The findings remained similar in the models without adjustment for BBD subtype (Supplementary Table 4). The results were also similar in the additional models with probit-transformation for tenascin (data not shown). No associations were found in the secondary analysis among 555 premenopausal women (Supplementary Table 5); the association of continuous cumulative average alcohol with TNC was no longer significant (*p* = 0.23) likely due to a smaller number of premenopausal women.

**Table 2:** Association of alcohol use with expression of stromal markers (log-transformed) in benign breast biopsy samples (beta coefficients and 95% Confidence Intervals)^a^.

Alcohol use	*N*	αSMA	TNC	FAP	s100a6	MMP14
Recent alcohol (at BBD)						
Continuous per 11 g or 1 drink	664	0.03 (−0.06, 0.13)	0.36 (−0.18, 0.89)	0.16 (−0.18, 0.49)	−0.05 (−0.25, 0.14)	0.14 (−0.22, 0.50)
Drinks per day						
Non-drinker	239	Reference	Reference	Reference	Reference	Reference
<1	356	−0.03 (−0.15, 0.09)	0.32 (−0.34, 0.98)	0.30 (−0.11, 0.71)	0.01 (−0.23, 0.25)	0.18 (−0.27, 0.62)
1–< 2	52	0.03 (−0.20, 0.25)	0.02 (−1.20, 1.24)	0.44 (−0.32, 1.19)	−0.12 (−0.57, 0.32)	0.08 (−0.73, 0.90)
≥2	17	0.17 (−0.20, 0.54)	1.59 (−0.41, 3.59)	0.62 (−0.63, 1.86)	−0.09 (−0.82, 0.65)	0.67 (−0.66, 2.00)
*p*-trend	664	0.36	0.26	0.21	0.61	0.43
Drinks per day						
Non-drinker	239	Reference	Reference	Reference	Reference	Reference
<1	356	−0.03 (−0.15, 0.09)	0.32 (−0.34, 0.98)	0.30 (−0.11, 0.71)	0.01 (−0.23, 0.25)	0.17 (−0.27, 0.62)
≥1	69	0.06 (−0.14, 0.26)	0.40 (−0.69, 1.50)	0.48 (−0.20, 1.16)	−0.11 (−0.51, 0.29)	0.23 (−0.50, 0.95)
*p*-trend	664	0.51	0.50	0.19	0.56	0.57
Cumulative average						
Continuous per 11 g or 1 drink	683	0.01 (−0.11, 0.12)	0.59 (−0.03, 1.22)	0.09 (−0.29, 0.48)	−0.06 (−0.29, 0.16)	0.06 (−0.35, 0.48)
Drinks per day						
Non-drinker	150	Reference	Reference	Reference	Reference	Reference
<1	477	0.10 (−0.04, 0.23)	0.23 (−0.51, 0.97)	0.24 (−0.22, 0.70)	0.07 (−0.20, 0.34)	0.36 (−0.13, 0.85)
1–< 2	43	0.17 (−0.08, 0.42)	0.27 (−1.11, 1.64)	0.80 (−0.05, 1.65)	0.11 (−0.39, 0.62)	0.35 (−0.55, 1.26)
≥2	13	0.12 (−0.30, 0.54)	2.50 (0.20, 4.80)	0.09 (−1.32, 1.50)	−0.45 (−1.29, 0.39)	0.32 (−1.20, 1.83)
*p*-trend	683	0.33	0.11	0.24	0.58	0.64
Drinks per day						
Non-drinker	150	Reference	Reference	Reference	Reference	Reference
<1	477	0.10 (−0.04, 0.23)	0.23 (−0.51, 0.98)	0.24 (−0.22, 0.70)	0.07 (−0.20, 0.34)	0.36 (−0.13, 0.85)
≥1	56	0.16 (−0.07, 0.39)	0.79 (−0.46, 2.03)	0.64 (−0.13, 1.40)	−0.02 (−0.48, 0.44)	0.35 (−0.48, 1.17)
*p*-trend	683	0.28	0.24	0.14	0.82	0.62

*αSMA* alpha-smooth muscle actin, *BBD* benign breast disease, *CI* confidence interval, *FAP* fibroblast activation protein, *MMP14* matrix metalloproteinase, *s100a6* calcyclin, *TNC* tenascin-C.

^a^Adjusted for age (continuous), BMI (continuous), a family history of breast cancer (Yes/No), menopausal status/postmenopausal hormone use (premenopausal, postmenopausal/no hormones, postmenopausal/past hormones, postmenopausal/current hormones, postmenopausal/unknown hormone use status), age at menarche ( < 12, 12, 13, >13, unknown), combined parity/age at first birth (parous with first birth before age 25, parous with first birth at or after age 25, nulliparous, unknown), and BBD category (non-proliferative, proliferative without atypia, and proliferative with atypia).

## Discussion

In this study of 683 cancer-free women with biopsy-confirmed incident BBD in the NHS II cohort, we examined the associations between recent and cumulative average alcohol consumption and the expression of stromal markers αSMA, TNC, FAP, s100a6, and MMP14. We found suggestive positive associations of cumulative average alcohol consumption with TNC expression.

Alcohol consumption is a well-established risk factor for breast cancer, with numerous studies showing a positive, dose-dependent association [1–3]. The International Agency for Research on Cancer (IARC) classifies alcohol as a group 1 carcinogen, with causal associations for breast cancer reported by the U.S. Surgeon General [8]. Previously suggested mechanisms underlying these associations include DNA damage caused by acetaldehyde, oxidative stress and promotion of inflammation [9, 10]. Additionally, alcohol consumption may elevate oestrogens (both circulating and in the breast tissue) via increases in aromatase activity [2, 9]. A recent study also suggested that alcohol may influence breast tissue composition [33]. Our findings of positive associations of cumulative average alcohol consumption with TNC expression suggest that alcohol can potentially influence normal breast fibroblasts. While all the selected markers for this study are associated with fibroblast activation, ECM remodelling, and tumour progression, unlike the rest of the markers that are localised in the cellular plasma, membrane or nucleus, TNC is secreted into ECM [34]. Previous studies in the breast tumours suggest that TNC contributes to carcinogenesis by promoting epithelial–mesenchymal transition (EMT), stem-cell signalling, and immune-suppressive environment and, as the result, enhancing cell motility, angiogenesis, invasion, and metastatic spread [35–37]. TNC expression in breast tumours has been linked to poorer patient outcomes and more aggressive tumour characteristics [38]. Prior reports showed expression of TNC in breast tumour and, to a much lesser degree, in adjacent normal tissue [36, 38]. Our findings now demonstrate that TNC may also be expressed in histologically normal breast tissue of cancer-free women, suggesting its potential involvement in the early stages of neoplastic transformation. If associations between alcohol and TNC are confirmed in subsequent studies, results may suggest that the earliest effects of alcohol on breast carcinogenesis may involve fibroblast activation in normal breast tissue. Given the complexity of the stromal environment, future studies should incorporate more relevant markers to comprehensively examine this complex relationship.

This is the first study to evaluate associations of alcohol consumption and the expression of several stromal markers in cancer-free women. The study used the data and breast biopsy samples from the Nurses’ Health Study II, a large, prospective cohort study with nearly 4 decades of follow-up and detailed information on BBD status and established breast cancer risk factors. Despite these strengths, several limitations should be noted. Biopsy samples were retrieved from a single region of breast tissue, which may limit the generalisability of findings to the entire breast. However, previous NHS studies have demonstrated that this sampling technique allows identification of markers associated with BCa risk, as well as investigating their associations with various breast cancer risk factors [39–46]. Although alcohol intake was assessed prospectively, self-reported consumption may be subject to misclassification. Nonetheless, prior validation studies have demonstrated high reproducibility and validity of alcohol data collected by the FFQ, with strong correlations between reported alcohol intake on FFQ and multiple-week diet records (*r* = 0.90), as well as repeated FFQs administered 4 years apart (*r* = 0.84) [47, 48]. Therefore, the FFQ remains a reliable tool for measuring long-term alcohol consumption. It should be noted that in our sample, only 18.7% of women consumed ≥11 g (≥1 drink) of alcohol per day at the time of the biopsy, and only 18.2% reported an average cumulative alcohol consumption ≥11 g (≥1 drink), limiting our ability to examine the effect of heavy drinking definitively. Finally, timing of exposure may also play a role, as adolescence is considered a window of susceptibility for breast carcinogenesis and early-life exposures can have long-lasting effects on subsequent breast cancer risk [2]. Some previous studies have demonstrated a positive association of adolescent alcohol consumption with the risk of proliferative BBD [49, 50], higher estradiol levels [51] and breast cancer [52, 53]. Previous studies that examined associations of early-lifer alcohol consumption as well as other breast cancer risk factors with breast cancer risk had varying definitions of the adolescent period (ranging between ages 10–20 years). In the context of breast cancer risk, adolescence could be defined as the period from the onset of puberty to the completion of breast development (around the age of 17–18 years). Additionally, the time between menarche and the first full-time pregnancy is of particular interest as the breast tissue remains undifferentiated and more susceptible to various influences during this period. In our study sample, data on adolescent alcohol consumption (as reported for the ages between 15 and 17 in NHSII) were available only for 444 (65%) participants, and among those, 415 reported no alcohol consumption during adolescence; thus, this association could not be evaluated.

In conclusion, our findings provide the first evidence of suggestive associations of alcohol consumption with TNC expression in histologically normal breast tissue. Future studies are warranted to confirm these findings and expand the set of relevant stromal markers to better capture the potential complexity of alcohol’s influence on stromal-epithelial interactions. Additionally, investigations into the associations of alcohol consumption during adolescence, as well as before the first full-term pregnancy, with stromal marker expression may help to better understand the biological mechanisms linking early-life alcohol consumption to breast cancer risk.

## Supplementary information

Supplementary Tables 1-5

Supplementary figure 1 and 2.

## Data Availability

The data that support the findings of this study are available from the Nurses’ Health Studies; they are not publicly available. Investigators interested in using the data can request access, and feasibility will be discussed at an investigator’s meeting. Limits are not placed on scientific questions or methods, and there is no requirement for co-authorship. Additional data sharing information and policy details can be accessed at http://www.nurseshealthstudy.org/researchers.

## References

[1] Jung S, Wang M, Anderson K, Baglietto L, Bergkvist L, Bernstein L, et al. Alcohol consumption and breast cancer risk by estrogen receptor status: in a pooled analysis of 20 studies. Int J Epidemiol. 2015;45:916–28. 26320033 10.1093/ije/dyv156 PMC5005939

[2] Liu Y, Nguyen N, Colditz GA. Links between alcohol consumption and breast cancer: a look at the evidence. Womens Health. 2015;11:65–77. 10.2217/whe.14.62 PMC4299758 25581056

[3] Sohi I, Rehm J, Saab M, Virmani L, Franklin A, Sánchez G, et al. Alcoholic beverage consumption and female breast cancer risk: a systematic review and meta-analysis of prospective cohort studies. Alcohol Clin Exp Res. 2024;48:2222–41. 10.1111/acer.15493 PMC11629438 39581746

[4] Boyle P, Boffetta P. Alcohol consumption and breast cancer risk. Breast Cancer Res. 2009;11:S3. 20030878 10.1186/bcr2422 PMC2797683

[5] Hirko KA, Chen WY, Willett WC, Rosner BA, Hankinson SE, Beck AH, et al. Alcohol consumption and risk of breast cancer by molecular subtype: prospective analysis of the nurses’ health study after 26 years of follow-up. Int J Cancer. 2016;138:1094–101. 26384849 10.1002/ijc.29861 PMC4715769

[6] McDonald JA, Goyal A, Terry MB. Alcohol intake and breast cancer risk: weighing the overall evidence. Curr Breast Cancer Rep. 2013;5:208–21. 10.1007/s12609-013-0114-z PMC3832299 24265860

[7] Scoccianti C, Lauby-Secretan B, Bello P-Y, Chajes V, Romieu I. Female breast cancer and alcohol consumption: a review of the literature. Am J Prev Med. 2014;46:S16–25. 24512927 10.1016/j.amepre.2013.10.031

[8] Office of the Surgeon General. Alcohol and cancer risk: the U.S. Surgeon General’s Advisory. U.S. Department of Health and Human Services; https://www.hhs.gov/surgeongeneral/reports-and-publications/alcohol-cancer/index.html 2025.

[9] Rumgay H, Murphy N, Ferrari P, Soerjomataram I. Alcohol and cancer: epidemiology and biological mechanisms. Nutrients. 2021;13:3173. 34579050 10.3390/nu13093173 PMC8470184

[10] Fanfarillo F, Caronti B, Lucarelli M, Francati S, Tarani L, Ceccanti M, et al. Alcohol consumption and breast and ovarian cancer development: molecular pathways and mechanisms. Curr Issues Mol Biol. 2024;46:14438–52. 39727994 10.3390/cimb46120866 PMC11674816

[11] Flom JD, Ferris JS, Tehranifar P, Terry MB. Alcohol intake over the life course and mammographic density. Breast Cancer Res Treat. 2009;117:643–51. 19184416 10.1007/s10549-008-0302-0 PMC4072459

[12] Maskarinec G, Takata Y, Pagano I, Lurie G, Wilkens LR, Kolonel LN. Alcohol consumption and mammographic density in a multiethnic population. Int J Cancer. 2006;118:2579–83. 16380998 10.1002/ijc.21705

[13] Ziembicki S, Zhu J, Tse E, Martin LJ, Minkin S, Boyd NF. The association between alcohol consumption and breast density: a systematic review and meta-analysis. Cancer Epidemiol Biomark Prev. 2017;26:170–8. 10.1158/1055-9965.EPI-16-0522 27672053

[14] Edmonds CE, O’Brien SR, Conant EF. Mammographic breast density: current assessment methods, clinical implications, and future directions. Semin Ultrasound CT MRI. 2023;44:35–45. 10.1053/j.sult.2022.11.001 36792272

[15] Ironside AJ, J J. Stromal characteristics may hold the key to mammographic density: the evidence to date. Oncotarget. 2016;7:31550–62. 26784251 10.18632/oncotarget.6912 PMC5058777

[16] McCuaig R, Wu F, Dunn J, Rao S, Dahlstrom JE. The biological and clinical significance of stromal-epithelial interactions in breast cancer. Pathology. 2017;49:133–40. 28040198 10.1016/j.pathol.2016.10.009

[17] Plikus MV, Wang X, Sinha S, Forte E, Thompson SM, Herzog EL, et al. Fibroblasts: origins, definitions, and functions in health and disease. Cell. 2021;184:3852–72. 34297930 10.1016/j.cell.2021.06.024 PMC8566693

[18] Yang D, Liu J, Qian H, Zhuang Q. Cancer-associated fibroblasts: from basic science to anticancer therapy. Exp Mol Med. 2023;55:1322–32. 37394578 10.1038/s12276-023-01013-0 PMC10394065

[19] Yazhou C, Wenlv S, Weidong Z, Licun W. Clinicopathological significance of stromal myofibroblasts in invasive ductal carcinoma of the breast. Tumour Biol. 2004;25:290–5. 15627894 10.1159/000081394

[20] Toullec A, Gerald D, Despouy G, Bourachot B, Cardon M, Lefort S, et al. Oxidative stress promotes myofibroblast differentiation and tumour spreading. EMBO Mol Med. 2010;2:211–30. 20535745 10.1002/emmm.201000073 PMC3377319

[21] Yamashita M, Ogawa T, Zhang X, Hanamura N, Kashikura Y, Takamura M, et al. Role of stromal myofibroblasts in invasive breast cancer: stromal expression of alpha-smooth muscle actin correlates with worse clinical outcome. Breast Cancer. 2012;19:170–6. 20978953 10.1007/s12282-010-0234-5

[22] Tchou J, Zhang PJ, Bi Y, Satija C, Marjumdar R, Stephen TL, et al. Fibroblast activation protein expression by stromal cells and tumor-associated macrophages in human breast cancer. Hum Pathol. 2013;44:2549–57. 24074532 10.1016/j.humpath.2013.06.016 PMC4283499

[23] Heizmann CW, Fritz G, Schäfer BW. S100 proteins: structure, functions and pathology. Front Biosci. 2002;7:d1356–68. 11991838 10.2741/A846

[24] Cui MDH, Duan W, Wang X, Liu Y, Shi L, Zhang B. The relationship between cancer associated fibroblasts biomarkers and prognosis of breast cancer: a systematic review and meta-analysis. PeerJ. 2024;12:e16958. 38410801 10.7717/peerj.16958 PMC10896086

[25] O’Connell JT, Sugimoto H, Cooke VG, MacDonald BA, Mehta AI, LeBleu VS, et al. VEGF-A and Tenascin-C produced by S100A4+ stromal cells are important for metastatic colonization. Proc Natl Acad Sci USA. 2011;108:16002–7. 21911392 10.1073/pnas.1109493108 PMC3179047

[26] Chen N, Zhang G, Fu J, Wu Q. Matrix metalloproteinase-14 (MMP-14) downregulation inhibits esophageal squamous cell carcinoma cell migration, invasion, and proliferation. Thorac Cancer. 2020;11:3168–74. 32930509 10.1111/1759-7714.13636 PMC7606025

[27] Bao Y, Bertoia ML, Lenart EB, Stampfer MJ, Willett WC, Speizer FE, et al. Origin, methods, and evolution of the three nurses’ health studies. Am J Public Health. 2016;106:1573–81. 27459450 10.2105/AJPH.2016.303338 PMC4981810

[28] Yaghjyan L, Heng YJ, Baker GM, Murthy D, Mahoney MB, Rosner B, et al. Associations of stem cell markers in benign breast tissue with subsequent breast cancer risk. Am J Cancer Res. 2023;13:6280–9. 38187066 PMC10767353

[29] Yaghjyan L, Heng YJ, Baker GM, Bret-Mounet V, Murthy D, Mahoney MB, et al. Reliability of CD44, CD24, and ALDH1A1 immunohistochemical staining: pathologist assessment compared to quantitative image analysis. Front Med. 2022;9:1040061. 10.3389/fmed.2022.1040061 PMC9794585 36590957

[30] Yaghjyan L, Heng YJ, Sardella BR, Murthy D, Mahoney MB, Rosner B, et al. Associations of circulating insulin-like growth factor-1 and insulin-like growth factor binding protein-3 with the expression of stem cell markers in benign breast tissue. Breast Cancer Res. 2025;27:53. 40197284 10.1186/s13058-025-02002-z PMC11978140

[31] Willett W, Stampfer MJ, Bain C, Lipnick R, Speizer FE, Rosner B, et al. Cigarette smoking, relative weight, and menopause. Am J Epidemiol. 1983;117:651–8. 6859020 10.1093/oxfordjournals.aje.a113598

[32] Stampfer MJ, Willett WC, Colditz GA, Rosner B, Speizer FE, Hennekens CH. A prospective study of postmenopausal estrogen therapy and coronary heart disease. N Engl J Med. 1985;313:1044–9. 4047106 10.1056/NEJM198510243131703

[33] Yaghjyan L, Heng YJ, Baker GM, Rosner BA, Tamimi RM. Associations of alcohol consumption with breast tissue composition. Breast Cancer Res. 2023;25:33. 36998083 10.1186/s13058-023-01638-z PMC10061845

[34] Chiquet-Ehrismann R, Tucker RP. Tenascins and the importance of adhesion modulation. Cold Spring Harb Perspect Biol. 2011;3:a004960. 10.1101/cshperspect.a004960 PMC3101840 21441591

[35] Wawrzyniak DGM, Głodowicz P, Kuczyński K, Kuczyńska B, Fedoruk-Wyszomirska A, Rolle K. Down-regulation of tenascin-C inhibits breast cancer cell growth, migration and adhesion. PLoS ONE. 2020;15:e0237889. 32817625 10.1371/journal.pone.0237889 PMC7440653

[36] Nagaharu K, Zhang X, Yoshida T, Katoh D, Hanamura N, Kozuka Y, et al. Tenascin C induces epithelial-mesenchymal transition-like change accompanied by SRC activation and focal adhesion kinase phosphorylation in human breast cancer cells. Am J Pathol. 2011;178:754–63. 21281808 10.1016/j.ajpath.2010.10.015 PMC3069868

[37] Chiquet-Ehrismann R, Chiquet M. Tenascins: regulation and putative functions during pathological stress. J Pathol. 2003;200:488–99. 12845616 10.1002/path.1415

[38] Yang Z, Ni W, Cui C, Fang L, Xuan Y. Tenascin C is a prognostic determinant and potential cancer-associated fibroblasts marker for breast ductal carcinoma. Exp Mol Pathol. 2017;102:262–7. 28223108 10.1016/j.yexmp.2017.02.012

[39] Oh H, Eliassen AH, Wang M, Smith-Warner SA, Beck AH, Schnitt SJ, et al. Expression of estrogen receptor, progesterone receptor, and Ki67 in normal breast tissue in relation to subsequent risk of breast cancer. NPJ Breast Cancer. 2016;2:16032. 28111631 10.1038/npjbcancer.2016.32 PMC5243126

[40] Oh H, Eliassen AH, Beck AH, Rosner B, Schnitt SJ, Collins LC, et al. Breast cancer risk factors in relation to estrogen receptor, progesterone receptor, insulin-like growth factor-1 receptor, and Ki67 expression in normal breast tissue. NPJ Breast Cancer. 2017;3:39. 28979927 10.1038/s41523-017-0041-7 PMC5624935

[41] Yaghjyan L, Heng YJ, Baker GM, Bret-Mounet VC, Murthy D, Mahoney MB, et al. Associations of reproductive breast cancer risk factors with expression of stem cell markers in benign breast tissue. Front Oncol. 2024;14:1354094. 38577336 10.3389/fonc.2024.1354094 PMC10991780

[42] Rice MS, Tamimi RM, Connolly JL, Collins LC, Shen D, Pollak MN, et al. Insulin-like growth factor-1, insulin-like growth factor binding protein-3 and lobule type in the Nurses’ Health Study II. Breast Cancer Res. 2012;14:R44. 22414675 10.1186/bcr3141 PMC3446378

[43] Huh SJ, Oh H, Peterson MA, Almendro V, Hu R, Bowden M, et al. The proliferative activity of mammary epithelial cells in normal tissue predicts breast cancer risk in premenopausal women. Cancer Res. 2016;76:1926–34. 26941287 10.1158/0008-5472.CAN-15-1927 PMC4873436

[44] Tamimi RM, Colditz GA, Wang Y, Collins LC, Hu R, Rosner B, et al. Expression of IGF1R in normal breast tissue and subsequent risk of breast cancer. Breast Cancer Res Treat. 2011;128:243–50. 21197570 10.1007/s10549-010-1313-1 PMC3116083

[45] Oh H, Yaghjyan L, Heng YJ, Rosner B, Mahoney MB, Murthy D, et al. Associations of early-life and adult anthropometric measures with the expression of stem cell markers CD44, CD24, and ALDH1A1 in women with benign breast biopsies. Cancer Epidemiol Biomark Prev. 2024;33:933–43. 10.1158/1055-9965.EPI-23-1567 PMC11216865 38652503

[46] Ratcliffe M, Heng YJ, Baker GM, Rosner B, Tamimi RM, Yaghjyan L. Associations of alcohol consumption with expression of CD44, CD24, and ALDH1A1 stem cell markers in benign breast biopsy samples. Cancer Causes Control. 2025;36:1489–97. 40650797 10.1007/s10552-025-02034-y PMC12766974

[47] Giovannucci E, Colditz G, Stampfer MJ, Rimm EB, Litin L, Sampson L, et al. The assessment of alcohol consumption by a simple self-administered questionnaire. Am J Epidemiol. 1991;133:810–7. 2021148 10.1093/oxfordjournals.aje.a115960

[48] Del Boca FK, Darkes J. The validity of self-reports of alcohol consumption: state of the science and challenges for research. Addiction. 2003;98(Suppl 2):1–12. 14984237 10.1046/j.1359-6357.2003.00586.x

[49] Berkey CS, Willett WC, Frazier AL, Rosner B, Tamimi RM, Rockett HRH, et al. Prospective study of adolescent alcohol consumption and risk of benign breast disease in young women. Pediatrics. 2010;125:e1081–7. 20385629 10.1542/peds.2009-2347 PMC3075610

[50] Liu Y, Tamimi RM, Berkey CS, Willett WC, Collins LC, Schnitt SJ, et al. Intakes of alcohol and folate during adolescence and risk of proliferative benign breast disease. Pediatrics. 2012;129:e1192–8. 22492774 10.1542/peds.2011-2601 PMC3866773

[51] Martin CA, Mainous AG 3rd, Curry T, Martin D. Alcohol use in adolescent females: correlates with estradiol and testosterone. Am J Addict. 1999;8:9–14. 10189510 10.1080/105504999306036

[52] Dydjow-Bendek DA, Zagożdżon P. Early alcohol use initiation, obesity, not breastfeeding, and residence in a rural area as risk factors for breast cancer: a case-control study. Cancers. 2021;13:3925. 34439080 10.3390/cancers13163925 PMC8394787

[53] Liu Y, Colditz GA, Rosner B, Berkey CS, Collins LC, Schnitt SJ, et al. Alcohol intake between menarche and first pregnancy: a prospective study of breast cancer risk. J Natl Cancer Inst. 2013;105:1571–8. 23985142 10.1093/jnci/djt213 PMC3797023

