# Supplementary material for: Associations of alcohol use with expression of stromal markers in benign breast biopsy samples
Source: Br J Cancer. 2026 Jun 11;135(5):784–90. doi: 10.1038/s41416-026-03481-3 (PMC13478342; doi:10.1038/s41416-026-03481-3)
Supplement: Supplementary file 1 [file 41416_2026_3481_MOESM1_ESM.docx]

**Supplementary table 1. Distribution of percent positivity for stromal markers in normal terminal duct lobular units of study women**

|  | **Mean (Standard deviation)** | **Minimum** | **Maximum** |
| --- | --- | --- | --- |
| **Alpha-smooth muscle actin (αSMA)** | 9.79 (5.60) | 0 | 44.51 |
| **Tenascin (TNC)** | 0.77 (3.93) | 0 | 52.51 |
| **Fibroblast activation protein (FAP)** | 7.62 (9.70) | 0 | 79.19 |
| **Matrix metalloproteinase (MMP14)** | 9.99 (12.26) | 0 | 67.79 |
| **Calcyclin (s100a6)** | 21.46 (14.71) | 0 | 70.54 |

**Supplementary Table 2. Correlations among stromal markers in benign breast biopsy samples (Spearman Correlation Coefficients and p-values)**

|  | **Tenascin (TNC)** | **Fibroblast Activation Protein (FAP)** | **Calcyclin (s100a6)** | **Matrix metalloproteinase 14 (MMP14)** |
| --- | --- | --- | --- | --- |
| **Alpha-smooth muscle actin (α-SMA)** | 0.09 (0.02) | 0.12 (<0.01) | 0.12 (<0.01) | 0.11 (<0.01) |
| **Tenascin (TNC)** |  | 0.06 (0.12) | 0.07 (0.07) | 0.24 (<0.0001) |
| **Fibroblast Activation Protein (FAP)** |  |  | 0.48 (<0.0001) | 0.57 (<0.0001) |
| **Calcyclin (s100a6)** |  |  |  | 0.56 (<0.0001) |

**Supplementary Table 3. Age- and BMI-Adjusted Association of alcohol use with expression of stromal markers (log-transformed) in benign breast biopsy samples (beta coefficients and 95% Confidence Intervals)**

| **Alcohol use** | **N** | **αSMA** | **TNC** | **FAP** | **S100a6** | **MMP14** |
| --- | --- | --- | --- | --- | --- | --- |
| **Recent alcohol (at BBD)** |  |  |  |  |  |  |
| Continuous per 11g or 1 drink | 664 | 0.02 (-0.08, 0.12) | 0.43 (-0.11, 0.97) | 0.16 (-0.17, 0.49) | -0.04 (-0.24, 0.15) | 0.14 (-0.22, 0.50) |
| Drinks per day  non-drinker  <1  1-<2  ≥2  p-trend | 239  356  52  17  664 | Reference  -0.02 (-0.15, 0.10)  0.04 (-0.19, 0.26)  0.11 (-0.26, 0.48)  0.49 | Reference  0.31 (-0.36, 0.98)  0.08 (-1.15, 1.30)  1.79 (-0.21, 3.79)  0.18 | Reference  0.30 (-0.11, 0.71)  0.39 (-0.37, 1.15)  0.66 (-0.58, 1.90)  0.22 | Reference  0.04 (-0.21, 0.28)  -0.10 (-0.55, 0.34)  -0.04 (-0.77, 0.69)  0.70 | Reference  0.19 (-0.25, 0.64)  0.04 (-0.78, 0.86)  0.76 (-0.57, 2.10)  0.41 |
| Drinks per day  non-drinker  <1  ≥1  p-trend | 239  356  69  664 | Reference  -0.02 (-0.15, 0.10)  0.05 (-0.15, 0.26)  0.57 | Reference  0.31 (-0.36, 0.98)  0.50 (-0.60, 1.60)  0.40 | Reference  0.30 (-0.11, 0.71)  0.46 (-0.22, 1.13)  0.21 | Reference  0.04 (-0.21, 0.28)  -0.09 (-0.49, 0.31)  0.65 | Reference  0.19 (-0.25, 0.64)  0.22 (-0.52, 0.95)  0.60 |
| **Cumulative average** |  |  |  |  |  |  |
| Continuous per 11g or 1 drink | 683 | -0.37x10^-2^ (-0.12, 0.11) | 0.69 (0.07, 1.32) | 0.07 (-0.31, 0.45) | -0.06 (-0.29, 0.17) | 0.04 (-0.37, 0.45) |
| Drinks per day  non-drinker  <1  1-<2  ≥2  p-trend | 150  477  43  13  683 | Reference  0.10 (-0.03, 0.24)  0.15 (-0.11, 0.40)  0.12 (-0.30, 0.55)  0.40 | Reference  0.16 (-0.59, 0.90)  0.30 (-1.08, 1.68)  2.65 (0.35, 4.95)  0.07 | Reference  0.25 (-0.21, 0.70)  0.72 (-0.12, 1.57)  0.08 (-1.33, 1.50)  0.30 | Reference  0.09 (-0.18, 0.36)  0.12 (-0.38, 0.63)  -0.40 (-1.24, 0.44)  0.63 | Reference  0.38 (-0.11, 0.87)  0.39 (-0.53, 1.30)  0.18 (-1.34, 1.70)  0.72 |
| Drinks per day  non-drinker  <1  ≥1  p-trend | 150  477  56  683 | Reference  0.10 (-0.03, 0.24)  0.14 (-0.09, 0.37)  0.36 | Reference  0.16 (-0.59, 0.91)  0.85 (-0.41, 2.10)  0.18 | Reference  0.25 (-0.21, 0.70)  0.58 (-0.19, 1.34)  0.19 | Reference  0.09 (-0.19, 0.36)  0.15x10^-2^ (-0.45, 0.46)  0.86 | Reference  0.38 (-0.11, 0.87)  0.34 (-0.49, 1.16)  0.65 |

**Abbreviations:** αSMA - alpha-smooth muscle actin; BBD – benign breast disease; CI- confidence interval; FAP- fibroblast activation protein; MMP14- matrix metalloproteinase; s100a6 – calcyclin; TNC - tenascin-C

**Supplementary Table 4. Association of alcohol use with expression of stromal markers (log-transformed) in benign breast biopsy samples, without adjustment for BBD subtype ^a^**

| **Alcohol use** | **N** | **αSMA** | **N** | **TNC** | **N** | **FAP** | **N** | **s100a6** | **N** | **MMP14** |
| --- | --- | --- | --- | --- | --- | --- | --- | --- | --- | --- |
| **Recent alcohol (at BBD)** |  |  |  |  |  |  |  |  |  |  |
| Continuous per 11g or 1 drink | 664 | 0.03 (-0.06, 0.13) | 664 | 0.35 (-0.18, 0.89) | 664 | 0.16 (-0.18, 0.49) | 664 | -0.05 (-0.25, 0.14) | 664 | 0.14 (-0.22, 0.50) |
| Drinks per day  non-drinker  <1  1-<2  ≥2  p-trend | 239  356  52  17 | Reference  -0.03 (-0.15, 0.09)  0.03 (-0.19, 0.26)  0.16 (-0.21, 0.53)  0.38 | 239  356  52  17 | Reference  0.31 (-0.35, 0.98)  0.07 (-1.15, 1.29)  1.49 (-0.52, 3.50)  0.28 | 239  356  52  17 | Reference  0.31 (-0.11, 0.72)  0.41 (-0.35, 1.16)  0.67 (-0.58, 1.91)  0.21 | 239  356  52  17 | Reference   0.01 (-0.23, 0.26)   -0.13 (-0.58, 0.32)  -0.08 (-0.82, 0.65)  0.60 | 239  356  52  17 | Reference  0.19 (-0.26, 0.63)  0.03 (-0.78, 0.84)  0.73 (-0.61, 2.06)  0.44 |
| Drinks per day  non-drinker  <1  ≥1  p-trend | 239  356  69 | Reference  -0.03 (-0.15, 0.09)  0.06 (-0.14, 0.26)  0.51 | 239  356  69 | Reference  0.31 (-0.36, 0.97)  0.41 (-0.69, 1.51)  0.77 | 239  356  69 | Reference  0.31 (-0.11, 0.72)  0.47 (-0.21, 1.15)  0.20 | 239  356  69 | Reference   0.01 (-0.23, 0.26)   -0.12 (-0.52, 0.28)  0.55 | 239  356  69 | Reference  0.19 (-0.26, 0.63)  0.20 (-0.53, 0.93)  0.63 |
| **Cumulative average** |  |  |  |  |  |  |  |  |  |  |
| Continuous per 11g or 1 drink | 683 | 0.01 (-0.11, 0.12) | 683 | 0.60 (-0.03, 1.23) | 683 | 0.07 (-0.31, 0.46) | 683 | -0.07 (-0.30, 0.15) | 683 | 0.02 (-0.39, 0.43) |
| Drinks per day  non-drinker  <1  1-<2  ≥2  p-trend | 150  477  43  13 | Reference  0.10 (-0.04, 0.23)  0.17 (-0.09, 0.42)  0.11 (-0.31, 0.54)  0.34 | 150  477  43  13 | Reference  0.21 (-0.53, 0.96)  0.25 (-1.13, 1.63)  2.48 (0.18, 4.78)  0.11 | 150  477  43  13 | Reference  0.25 (-0.21, 0.71)  0.79 (-0.06, 1.64)  0.05 (-1.36, 1.46)  0.27 | 150  477  43  13 | Reference  0.07 (-0.20, 0.34)  0.10 (-0.40, 0.60)  -0.48 (-1.32, 0.36)  0.53 | 150  477  43  13 | Reference  0.37 (-0.12, 0.86)  0.31 (-0.60, 1.22)  0.19 (-1.33, 1.71)  0.80 |
| Drinks per day  non-drinker  <1  ≥1  p-trend | 150  477  56 | Reference  0.10 (-0.04, 0.23)  0.15 (-0.07, 0.38)  0.29 | 150  477  56 | Reference  0.22 (-0.53, 0.96)  0.77 (-0.48, 2.02)  0.25 | 150  477  56 | Reference  0.25 (-0.21, 0.71)  0.62 (-0.15, 1.38)  0.15 | 150  477  56 | Reference  0.07 (-0.20, 0.34)  -0.04 (-0.49, 0.42)  0.76 | 150  477  56 | Reference  0.37 (-0.12, 0.86)  0.28 (-0.55, 1.10)  0.76 |

**Abbreviations:** αSMA - alpha-smooth muscle actin; BBD – benign breast disease; CI- confidence interval; FAP- fibroblast activation protein; MMP14- matrix metalloproteinase; s100a6 – calcyclin; TNC - tenascin-C

^a^ Adjusted for age (continuous), BMI (continuous), a family history of breast cancer (Yes/No), menopausal status/postmenopausal hormone use (premenopausal, postmenopausal/no hormones, postmenopausal/past hormones, postmenopausal/current hormones, postmenopausal/unknown hormone use status), age at menarche (<12, 12, 13, >13, unknown), and combined parity/age at first birth (parous with first birth before age 25, parous with first birth at or after age 25, nulliparous, unknown)

**Supplementary Table 5. Association of alcohol use with expression of stromal markers (log-transformed) in benign breast biopsy samples among premenopausal women (beta coefficients and 95% Confidence Intervals) ^a^**

| **Alcohol use** | **N** | **αSMA** | **TNC** | **FAP** | **s100a6** | **MMP14** |
| --- | --- | --- | --- | --- | --- | --- |
| **Recent alcohol (at BBD)** |  |  |  |  |  |  |
| Continuous per 11g or 1 drink | 541 | -0.05 (-0.14, 0.04) | 0.16 (-0.47, 0.80) | 0.15 (-0.24, 0.53) | -0.03 (-0.25, 0.19) | 0.19 (-0.21, 0.60) |
| Drinks per day  non-drinker  <1  1-<2  ≥2  p-trend | 190  300  40  11  541 | Reference  -0.11 (-0.22, -0.01)  -0.09 (-0.29, 0.12)  -0.15 (-0.51, 0.22)  0.36 | Reference  0.40 (-0.33, 1.14)  0.24 (-1.16, 1.62)  0.22 (-2.27, 2.71)  0.81 | Reference  0.31 (-0.14, 0.76)  0.58 (-0.27, 1.43)  0.57 (-0.95, 2.09)  0.20 | Reference  0.11(-0.15, 0.36)  -0.07 (-0.56, 0.41)  0.01 (-0.86, 0.88)  0.81 | Reference  0.16 (-0.31, 0.63)  0.40 (-0.49, 1.29)  0.82 (-0.78, 2.41)  0.22 |
| Drinks per day  non-drinker  <1  ≥1  p-trend | 190  300  51  541 | Reference  -0.11 (-0.22, -0.01)  -0.10 (-0.28, 0.09)  0.33 | Reference  0.40 (-0.33, 1.14)  0.23 (-1.03, 1.50)  0.75 | Reference  0.31 (-0.14, 0.76)  0.58 (-0.19, 1.35)  0.15 | Reference  0.11 (-0.15, 0.36)  -0.05 (-0.50, 0.39)  0.78 | Reference  0.16 (-0.31, 0.63)  0.48 (-0.33, 1.30)  0.25 |
| **Cumulative average** |  |  |  |  |  |  |
| Continuous per 11g or 1 drink | 555 | -0.06 (-0.16, 0.05) | 0.43 (-0.28, 1.14) | 0.05 (-0.38, 0.48) | -0.06 (-0.31, 0.19) | 0.05 (-0.40, 0.50) |
| Drinks per day  non-drinker  <1  1-<2  ≥2  p-trend | 114  398  35  8  555 | Reference  0.04 (-0.09, 0.16)  0.06 (-0.17, 0.29)  -0.11 (-0.54, 0.33)  0.97 | Reference  0.33 (-0.52, 1.17)  0.39 (-1.14, 1.92)  2.33 (-0.59, 5.25)  0.23 | Reference  0.32 (-0.20, 0.83)  0.73 (-0.20, 1.66)  -0.33 (-2.11, 1.44)  0.51 | Reference  0.22 (-0.08, 0.52)  0.21 (-0.33, 0.74)  -0.71 (-1.74, 0.31)  0.50 | Reference  0.45 (-0.08, 0.99)  0.54 (-0.43, 1.51)  -0.30 (-2.16, 1.56)  0.83 |
| Drinks per day  non-drinker  <1  ≥1  p-trend | 114  398  43  555 | Reference  0.04 (-0.09, 0.16)  0.03 (-0.18, 0.24)  0.88 | Reference  0.32 (-0.52, 1.16)  0.75 (-0.67, 2.17)  0.36 | Reference  0.32 (-0.19, 0.83)  0.53 (-0.33, 1.40)  0.33 | Reference  0.22 (-0.07, 0.52)  0.04 (-0.46, 0.54)  0.80 | Reference  0.45 (-0.08, 0.99)  0.38 (-0.52, 1.29)  0.66 |

**Abbreviations:** αSMA - alpha-smooth muscle actin; BBD – benign breast disease; CI- confidence interval; FAP- fibroblast activation protein; MMP14- matrix metalloproteinase; s100a6 – calcyclin; TNC - tenascin-C

^a^ Adjusted for age (continuous), BMI (continuous), a family history of breast cancer (Yes/No), age at menarche (<12, 12, 13, >13, unknown), combined parity/age at first birth (parous with first birth before age 25, parous with first birth at or after age 25, nulliparous, unknown), and BBD category (non-proliferative, proliferative without atypia, and proliferative with atypia)
