# Supplementary material for: Associations of alcohol use with expression of stromal markers in benign breast biopsy samples
Source: Br J Cancer. 2026 Jun 11;135(5):784–90. doi: 10.1038/s41416-026-03481-3 (PMC13478342; doi:10.1038/s41416-026-03481-3)
Supplement: Supplementary file 2 [file 41416_2026_3481_MOESM2_ESM.docx]

**Supplementary figure 1. Distribution of tenascin-C staining categories by cumulative average alcohol**

Percentage

| Cumulative average alcohol | 0 | >0-<1 | ≥1-<10 | ≥10 |
| --- | --- | --- | --- | --- |
| None (n=150) | 71.9 | 16.7 | 8.8 | 2.6 |
| <11 g/day (n=478) | 67.1 | 23.4 | 8.5 | 1 |
| ≥11 g/day (n=56) | 67.8 | 25.6 | 9.3 | 2.3 |

**Supplementary figure 2. Distribution of log-transformed tenascin-C by cumulative average drinking in the study** **sample.**
